# Oropharyngeal dysphagia telerehabilitation in the Intensive Care Unit for COVID-19: a case report

**DOI:** 10.1590/2317-1782/20212021023

**Published:** 2022-04-01

**Authors:** Shigeto Soyama, Tomoo Mano, Akira Kido

**Affiliations:** 1 Department of Rehabilitation Medicine, Nara Medical University - Kashihara, Japan.; 2 Department of Neurology, Nara Medical University - Kashihara, Japan.

**Keywords:** Coronavirus Infections, COVID-19, Oropharyngeal Dysphagia, Swallowing Disorders, Rehabilitation, Telerehabilitation, Ventilation

## Abstract

The face-to-face assessment of and training for dysphagia are considered aerosol-generating procedures, and thus are contraindicated for patients who are positive or suspected of having severe acute respiratory syndrome coronavirus 2 infection. Considering the extremely infectious nature of the virus, transmission to other individuals during rehabilitation is possible. Some patients in the intensive care unit and those who are on endotracheal intubation and mechanical ventilation often have dysphagia. Therefore, assessment and training for oropharyngeal dysphagia are provided by rehabilitation professionals to restore normal feeding before patient discharged. Thus, we aimed to explore the advantages of telerehabilitation in dysphagia management during the coronavirus disease 2019 (COVID-19) pandemic. An infected 50-year-old man admitted to the hospital underwent extracorporeal membrane oxygenation rescue therapy and tracheostomy. Upon gradual respiratory status stabilization, extracorporeal membrane oxygenation therapy was discontinued, and he was weaned off the ventilator. He had difficulty swallowing and coughed after attempting to drink fluids. We considered the application of telerehabilitation for managing dysphagia while minimizing the risk of infection and usage of personal protective equipment. A videoconferencing software on a tablet device provided contactless telerehabilitation, thus reducing the risk of infection and preventing personal protective equipment shortage. Moreover, it facilitates discussion on the issues related to the evaluation of oropharyngeal dysphagia telerehabilitation. We highlight important considerations for the application of telerehabilitation in the assessment and treatment of dysphagia during the COVID-19 pandemic.

## INTRODUCTION

The widespread cases of severe acute respiratory syndrome coronavirus 2 (SARS-CoV-2) caused by coronavirus disease 2019 (COVID-19) has created a significant impact as a pandemic. Pneumonia due to COVID-19 may be characterized by hypoxic respiratory insufficiency. Patients with declining oxygen saturation levels are generally provided respiratory support, including endotracheal intubation and invasive mechanical ventilation; however, these modalities increase the risk of dysphagia. Furthermore, some cases can lead to post-intubation dysphagia^(1)^. Therefore, clinical assessment and examination of swallowing, oral care, and targeted swallowing training by specialized rehabilitation professionals are necessary to restore normal feeding before patients can be discharged^(2)^. Early intervention is crucial for preventing malnutrition and pneumonia. However, a direct assessment of the swallowing function of patients who test positive for or are suspected of having SARS-CoV-2 is not recommended. Currently, person-to-person transmission routes of SARS-CoV-2 include direct transmission; droplet transmission through coughing, sneezing, and talking; and contact transmission through contact with oral, nasal, and eye mucous membranes^(3)^. This can be associated with the risk of aerosol-particle generation, as in the case of physiotherapy for airway clearance and sputum collection^(4)^. Rehabilitation professionals who conduct swallowing therapy should screen for COVID-19 and carefully assess the urgency of interventions. Direct medical care encounters gradually increase the potential risk of nosocomial transmission. This in turn can be accredited to personal distancing and personal protective equipment (PPE) shortage. The virus can be transmitted to other patients from medical staff who are asymptomatic or pre-symptomatic carriers through face-to-face rehabilitation. Additionally, this may cause nosocomial infections, thus forming in-hospital clusters. SARS-CoV-2 is transmitted through droplets and direct contact. Furthermore, inhalation of aerosol particles increases the risk of infection. Examination of the oral cavity, pharyngeal region, and larynx of patients poses a higher risk of infection^(5)^. Tasks related to food intake and swallowing rehabilitation, such as functional assessments and training, involve contact, which can lead to the generation of droplets and aerosolized particles. Therefore, a nurse-mediated assessment of the risk of dysphagia is particularly appropriate during the unavailability of rehabilitation professionals. This may also be applicable during the current pandemic. Recent studies reported the validity and safety of oropharyngeal dysphagia telerehabilitation in patients with varying levels of dysphagia^(6)^. Oropharyngeal dysphagia telerehabilitation was modified to optimize patient support improved in term of operational processes for its use in clinical practice. This intensive care unit (ICU) telepractice service appears promising for dysphagia management owing to its high service and satisfaction^(7)^.

## CASE PRESENTATION

The study was approved by the institutional ethics committee of Nara Medical University under evaluation report no. 2581, and written informed consent was obtained from the patient.

**Figure 1:**
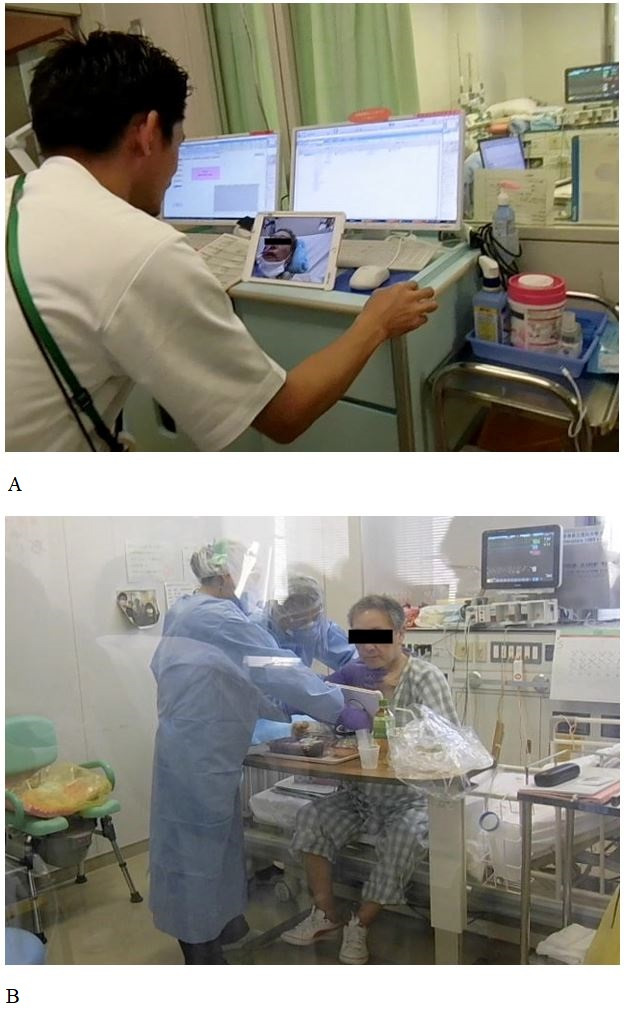
(A) Swallowing function assessment under the guidance of rehabilitation professionals for contactless execution of telerehabilitation from outside the patient’s ICU room; (B) Rehabilitation professionals in complete personal protective equipment directly performing assessment and training procedures in ICU room

A 50-year-old man with COVID-19 who had bilateral opacities throughout the lung fields with predominance in the lower lung lobes was admitted to the hospital. After receiving medication, his cough, dyspnea, and fatigue did not improve; therefore, intubation was initiated, and he was transferred to the ICU for further management. Subsequently, extracorporeal membrane oxygenation (ECMO) rescue therapy and tracheostomy were performed. As his respiratory status gradually stabilized, ECMO was discontinued, and he started to wean off the ventilator. The patient experienced swallowing difficulties and cough after attempting to drink fluids. We considered the application of telerehabilitation for dysphagia while minimizing the risk of infection and usage of PPE based on previous reports and guidelines. A videoconferencing software was used to perform the functional assessment of swallowing. A primary care nurse equipped in complete protective gear entered the patient’s room, while the rehabilitation professionals were stationed outside the ICU room. We used FaceTime® on a tablet device to provide remote support to the patient. The patient’s dysphagia status was assessed using a part of the Clinical Swallowing Examination^(8)^. The primary care nurse demonstrated the correct method of feeding to the patient after obtaining instructions from the rehabilitation professionals. The nurse observed the motion of oral cavity and larynx according to the Clinical Swallowing Examination protocol (Figure 1A) using the camera of the tablet device. Concurrently, the rehabilitation professionals assessed the oromotor and laryngeal domains of the Clinical Swallowing Examination (Table 1). The rehabilitation professionals provided direct instructions to the patient through verbal communication. Despite the patient wearing a mask, repetitive saliva swallowing tests (RSSTs) and cervical auscultation were performed (Figure 1B). This can be attributed to the low risk of infection related to coughing and sputum. The rehabilitation professionals assessed the RSST, which revealed one time swallowing in 30s. We used the camera to assess the basic functions of the oral cavity and pharyngeal movements, and to conduct repetitive training of the gross-motor movements, which facilitated the exclusion of aerosol-generating procedures. The rehabilitation professionals performed direct assessment and training after the patient tested negative for SARS-CoV-2. There was no substantial difference between the telerehabilitation and face-to-face findings of the Clinical Swallowing Examination. The swallowing function improved to three times in 30s. and the patient reported high levels of satisfaction with oropharyngeal dysphagia telerehabilitation.

**Table 1:** Clinical Swallowing Examination across telerehabilitation and Face to Face assessed by rehabilitation professionals

CSE Items	telerehabilitation		Face To Face
	Initiate	Last	
General orientation and alertness			
Alertness	1	1	1
Comprehension	1	1	1
Oromotor examination and laryngeal function			
Cranial nerve V: jaw symmetry	1	1	1
Cranial verve VII			
Squeeze eyes	1	1	1
Raise eyebrows	1	1	1
Open/close mouth with resistance	1	1	1
Smile	1	1	1
Puff cheeks	2	1	1
Lip pucker	2	1	1
Smile-pucker	2	1	1
Cranial nerves IX and X			
“Ahh” nasality	N.A.	N.A.	N.A.
“Ahh” breath support	N.A.	N.A.	N.A.
“NeiPei”nasality	N.A.	N.A.	N.A.
Count nasality	N.A.	N.A.	N.A.
Count voice Quality	N.A.	N.A.	N.A.
Laryngeal elevation	3	1	2
Cough on cue	2	1	1
Clearing throat on cue	3	2	1
Soft palate function	2	1	1
Cranial nerve XII			
Tongue protrusion/retraction	2	1	1
Tongue protrusion/retraction with speed	2	1	1
Tongue tip up/down	2	1	1
Tongue tip up/down with speed	2	1	1
Licking lips	1	1	1
Licking lips with speed	2	1	1
Tongue in cheek	2	1	1
Oral cavity hygiene and dentition		1	1
Dentition	1	1	1
Dentures	1	1	1
Denture fitting	1	1	1
Hygiene	2	1	1
Oral sores/ulceration	1	1	1
Pooling of saliva/secretions	2	1	1
Food and fluid trials			
Fluids: anterior spillage	N.A.	N.A.	1
Fluids: oral pharyngeal transit	N.A.	N.A.	1
Fluids: delayed pharyngeal swallow	N.A.	N.A.	1
Fluids: no. of swallows	N.A.	N.A.	1
Fluids: Laryngeal elevation	N.A.	N.A.	1
Fluids: wet voice	N.A.	N.A.	1
Fluids: oral pooling/residue	N.A.	N.A.	1
Fluids: volitional cough	N.A.	N.A.	1
Fluids: clearing throat	N.A.	N.A.	1
Food: anterior pharyngeal transit	N.A.	N.A.	1
Food: delayed pharyngeal swallow	N.A.	N.A.	1
Food: no. of swallows	N.A.	N.A.	1
Food: laryngeal elevation	N.A.	N.A.	1
Food: wet voice	N.A.	N.A.	1
Food: oral pooling/residue	N.A.	N.A.	1
Food: volitional cough	N.A.	N.A.	1
Food: clearing throat	N.A.	N.A.	1
Clinical decisions and recommendations			
Oral/nonoral recommendation	2	1	1
Diet decision: fluids	3	1	1
Diet decision: food	3	2	2
DOSS	1	1	1
Need for feeding assistance	2	1	1
Need for oral care	3	1	1
Need for MBS	1	1	1
MBS urgency	1	1	1
Need for FEES	1	1	1
FEES urgency	1	1	1
Need for referral to other professionals	1	1	1
Need and urgency of SLP review	1	1	1

Caption: The score of Clinical Swallowing Examination; 1 = normal function; 2 = slight or mild reduction in function or task performance; 3 = moderate reduction in task performance, can complete tasks but with some difficulty; 4 = moderate to severe reduction in ability to complete tasks, attempts tasks but with difficulty; 5 = severe impairment, minimal ability to complete inability to complete task; N.A. = Not applicable; DOSS = dysphagia outcome and severity scale; MBS = modified barium swallow; FEES = fiberoptic endoscopic evaluation of swallowing

## DISCUSSION

Telerehabilitation is a tool that can be applied in rehabilitation medicine to conduct online clinical assessments^(9)^. Multiple benefits, including improved access, better time efficiency, greater client focus, enhanced caseload management, and cost efficiency, have been reported^(10)^. Furthermore, we detected an additional benefit of infection control. Telerehabilitation is implemented remotely without physical interactions between the patient and physician; thus, it could prevent the spread of infection to the physician. Additionally, the development of the videoconferencing tool often involves intensive, detail-oriented, and interactive assessments of medical professionals and patients, enabling the delivery of remote care with the same quality as that of face-to-face assessments^(11,12)^. It offers a high level of safety and can be used for early intervention. However, telerehabilitation for swallowing difficulties, including a contactless assessment and training via videoconferencing, has limitation. While microaspiration may have been overlooked, silent aspiration may also have been undetected. The case also described earlier has some limitations. The method was neither validated nor evidence-based, as it had been contrived to meet an urgent medical need. Therefore, a secured videoconferencing software and protocol of telerehabilitation systems, such as technology designed to optimize high-quality audiovisual during a real-time interaction, should be used. We could not evaluate the movement of the thyroid notch because a marker was not appropriately placed. Moreover, the patient involved in this case maintained a good level of consciousness, and thus understood how to use the tablet device. It is necessary to confirm the safety and efficacy in a large number of cases. We could not compare the efficacy of telerehabilitation with face-to-face therapy; hence, telerehabilitation cannot be strongly recommended, since its impact on the results of swallowing in the present case was not measured. However, telerehabilitation has been shown to be efficient resource not only for infection prevention but also for patient care required by rehabilitation specialists, enabling the same quality of care as that of face-to-face interventions. Moreover, this may aid in the assessment of several patients, specifically those living in regions where there is a shortage of qualified rehabilitation professionals^(9)^.

## FINAL COMMENTS

Currently, the assessment of all in-patients is essential, and discharging patients as early as possible is vital to reduce the rate of contamination, rendering speech therapy essential. A speech therapist should evaluate the performance of patients with clinical priorities, to enable early and safe discharge from the hospital^(13)^. There are a few reports of remote and acute implementation in the ICU. There is no significant difference between evaluations using telerehabilitation and face-to-face evaluations. Furthermore, enhancing knowledge and education regarding a detailed swallowing function evaluation method is important. Telerehabilitation is a valuable tool in the ICU, as it demonstrates the ease of performing swallowing rehabilitation during the COVID-19 pandemic. Hence, the aforementioned case supports the potential use of telerehabilitation as a viable clinical modality for dysphagia management in the future.
